# Hepatitis B Virus-X Downregulates Expression of Selenium Binding Protein 1

**DOI:** 10.3390/v12050565

**Published:** 2020-05-20

**Authors:** Young-Man Lee, Soojin Kim, Ran-Young Park, Yeon-Soo Kim

**Affiliations:** 1 Dasan Undergraduate College, Ajou University, Suwon 16499, Korea; lymbest@ajou.ac.kr; 2 Graduate School of New Drug Discovery & Development, Chungnam National University, Daejeon 34134, Korea; soojinskim86@gmail.com; 3 Department of Smart Food & Drugs, Inje University, Gimhae 50834, Korea; babidream@naver.com

**Keywords:** hepatitis B virus, X protein, selenium binding protein 1, hepatocellular carcinoma

## Abstract

Selenium binding protein 1 (SELENBP1) has been known to be reduced in various types cancer, and epigenetic change is shown to be likely to account for the reduction of *SELNEBP1* expression. With cDNA microarray comparative analysis, we found that SELENBP1 is markedly decreased in hepatitis B virus-X (HBx)-expressing cells. To clarify the effect of HBx on *SELENBP1* expression, we compared the expression levels of *SELENBP1* mRNA and protein by semi-quantitative RT-PCR, Northern blot, and Western blot. As expected, *SELENBP1* expression was shown to be reduced in cells expressing HBx, and reporter gene analysis showed that the *SELENBP1* promoter is repressed by HBx. In addition, the stepwise deletion of 5′ flanking promoter sequences resulted in a gradual decrease in basal promoter activity and inhibition of *SELENBP1* expression by HBx. Moreover, immunohistochemistry on tissue microarrays containing 60 pairs of human liver tissue showed decreased intensity of SELENBP1 in tumor tissues as compared with their matched non-tumor liver tissues. Taken together, our findings suggest that inhibition of *SELENBP1* expression by HBx might act as one of the causes in the development of hepatocellular carcinoma caused by HBV infection.

## 1. Introduction

Selenium (Se) has been recognized as an essential trace element exhibiting potent anti-carcinogenic properties. Selenium binding protein 1 (SBP1, SELENBP1, hSP56) was first found in mouse liver due to its ability to bind exogenously administered selenium [1,2]. It has been suggested that SELENBP1 plays a role in regulating protein trafficking and secretion, as was it shown to regulate vesicular transport in an intra-golgi transport cell-free assay in vitro [3]. It has been shown that TGF-beta could regulate the expression of *SELENBP1* in chronic allograft nephropathy [4]. SELENBP1 may be involved in anti-carcinogenic activities, such as growth regulation, reduction/oxidation modulation, and detoxification [5]. Reduced expressions of *SELENBP1* with poor prognosis were shown in various carcinomas, including colorectal carcinoma [6,7], ovarian cancer [8], breast cancer [9,10], gastric carcinoma [11], lung adenocarcinoma [12,13,14], prostate cancer [15], thyroid carcinoma [16], bronchial epithelial cancer [17], esophageal adenocarcinoma [18], malignant melanoma [19], and hepatocellular carcinoma [20,21,22]. However, its physiological role or the molecular mechanism in cancers are not clear at present.

Human hepatitis B virus (HBV) is a leading cause of hepatitis, cirrhosis, and hepatocellular carcinoma (HCC) [23]. Among the proteins encoded by the HBV genome, the X gene product (HBx) is essential for the viral replication [24] and has drawn considerable attention regarding its pleiotropic functions [25,26,27]. HBx can affect cell growth, transformation, and balance between pro-apototic and anti-apoptotic effects [28,29,30,31,32]. The mechanism of balancing or favoring these different outcomes is not known, but the level of HBx in livers might be one of the important determinants. HBx stimulates many different signal transduction pathways and interacts with the basal transcription machinery. It does not bind directly to DNA, but transactivates multiple DNA elements such as CREB/ATF, AP-1, NF-κB, and Egr 1-binding sites [33,34,35,36,37]. HBx also interacts with different cellular partners relevant to cell transformation, such as p53, DDB1, Crm1, and the proteasome complex [38,39,40]. HBx expression was found to be preferentially maintained in HCCs. Although HBx has no direct transforming activity, it may act as a co-factor in different models of liver carcinogenesis [41]. HBx protein upregulates the expression of *c-myc* which downregulates the expression of miR-16/15a [42]. The microRNAs act as tumor suppressor and inhibit cell proliferation, clonal formation, and anchorage-independent growth abilities during the initiation and progression of HCC [42]. In addition, Zhu et al. [43] found that HBx promoted the expression of AFP protein which is known to stimulate the expression of some protooncogenes in hepatocytes by the PI3K/AKT signaling pathway, resulting in the survival of the HBV-infected hepatocytes. Considering the up- or down-regulation of miR-16/15a and AFP, HBx may promote the malignant transformation of hepatocytes. HBx protein also promotes the proliferation of hepatoma cells. Wang et al. found that HBx downregulated tumor-suppressor *p16* gene by methylation of some CpG sites in the promoter region [44]. HBx protein activates the Wnt/β-catenin pathway which results in the acceleration of hepatoma cell proliferation [45]. In addition to the promotion of transformation and proliferation in the development of HCC, HBx protein has been known to be involved in anti-apoptosis [46,47], migration/metastasis of hepatoma cells [48], and stabilization of HBV DNA in hepatoma cells [49]. Taken together, as a key viral oncoprotein, HBx plays crucial roles in the development of HCC, whose primary role is to enhance the transformation of liver cell because of its activities on cell cycle regulation and signaling pathways. However, the actual impact of HBx in the development of liver cancer and its mechanism of action remain controversial.

In this study, we show that *SELENBP1* expression is reduced in HBx-expressing human cells and human liver tumors as compared with matched non-tumor (counterpart normal) liver tissues. Our findings suggest that *SELENBP1* downregulation by the HBx protein might be implicated in the development of HCC.

## 2. Materials and Methods 

### 2.1. Cell Culture

Chang V9, Chang X31, and Chang X34 cells derived from human HeLa [Chang liver] cells (ATCC CCL-13, U.S.A.) were gifted form Dr. H. Cho (Ajou University, Korea) and were maintained in Dulbecco’s modified Eagle’s medium (DMEM) containing 10% fetal bovine serum (FBS; Life Technologies, Carlsbad, CA, U.S.A.). The Chang V9 control cell line was established after transfection of the cells with pTRE, an empty control vector (Clontech, Mountain View, CA, U.S.A.). Chang X31 and Chang X34 cell lines were established after transfection of the cells with pTet-X [29]. Chang X31 and Chang X34 cells constitutively express influenza hemagglutinin (HA)-tagged HBx protein. HEK293 cells (ATCC CRL-1573, Manassas, VA, U.S.A.) were maintained in Dulbecco’s modified Eagle’s medium (DMEM) containing 10% fetal bovine serum (FBS; Life Technologies, Carlsbad, CA, U.S.A.).

### 2.2. Plasmid Construction

The 5′ flanking region of the *SELENBP1* promoter (−1584 to −34) was amplified by PCR from human genomic DNA. The PCR product was cloned into pGL2-Basic vector (Promega, Madison, WI, U.S.A.) to construct pGL2-SELENBP1/1584. The 1584-Lck was constructed by inserting both 5′-flanking regions of *SELENBP1* promoter from pGL2-SELENBP1/1584 and *Lck* (lymphocyte-specific protein tyrosine kinase) cDNA into pHYK [50]. Deletion constructs containing different lengths of the 5′-flanking sequences of the *SELENBP1* gene were generated by PCR amplification from the 1584-Lck plasmid. To express HBx transiently, the HA-tagged HBx gene was cloned into pRcCMV (Invitrogen, Waltham, NY, U.S.A.) to give pRcCMV-HBx.

### 2.3. DNA Transfection

For promoter assay, pMyk-eGFP [50], *SELENBP1* promoter-Lck reporter construct, and pRcCMV-HBx were transiently transfected into cells using Lipofectatime (Invitrogen). pcDNA3.1 was added to equalize the amount of DNA transfected in each well whenever necessary. Cells were harvested 48 h after transfection.

### 2.4. RT-PCR

Total RNA was extracted with the RNeasy Kit (Qiagen, Germanton, MD, U.S.A.) according to the manufacturer’s instruction. cDNA synthesis was performed for 1 h at 37 °C using 1 μg of total RNA, 100 pmol oligodT18, and 40 U reverse transcriptase (Promega). Linear amplification ranges for each gene were tested on the adjusted cDNA.

### 2.5. Northern Blotting

For Northern blot analysis, total RNA was separated by electrophoresis in a 1.5% formaldehyde agarose gel, and hybridization was performed using a digoxigenin-labelled probe (Roche, Basel, Switzerland). After hybridization with the probe, the blots were washed, mRNAs were then detected by an alkaline phosphatase-labeled antibody against digoxigenin (Roche) and visualized with autoradiography film.

### 2.6. Western Blotting

For Western blot analysis, equal amounts of cell or tissue lysates were resolved by SDS-PAGE, and Western blot analysis was performed with the following antibodies: mouse monoclonal anti-HA (Roche), mouse monoclonal anti-GFP (Santa Cruz Biotechnology, Dallas, TX, U.S.A.), mouse monoclonal anti-Lck (Santa Cruz), rabbit polyclonal anti-SELENBP1 (MBL International, Woburn, MA, U.S.A.), mouse monoclonal anti-β-actin (Sigma-Aldrich Corp, St. Louis, MO, U.S.A.), and a mouse anti-HBx monoclonal antibody (Chemicon, Temecula, CA, U.S.A.). After washing the membranes with T-TBS, they were further incubated with horseradish peroxidase-conjugated secondary antibody (Sigma). Immunoblots were revealed by autoradiography using the enhanced chemiluminescence (ECL) detection kit (Amersham Biosciences, Uppsala, Sweden) and exposed to X-ray films (Kodak, Augsberg, Germany).

### 2.7. Immunohistochemistry

Hepatocellular carcinoma tissue microarray (TMA) and tissue samples were provided from the Liver Cancer Tissue Bank (Yonsei University, Korea) and approved by the Liver Cancer Tissue Bank review committee (IRB #GB-2007-003). Sections were deparaffinized in xylene and rehydrated with a descending series of ethanol. For antigen retrieval, sections were incubated in boiling citrate buffer (10 mM, pH 6) for 40 min. Endogenous peroxidase activity was quenched using 3% H_2_O_2_ for 20 min. Sections were then blocked with PBS containing 5% bovine serum albumin and were subsequently incubated with anti-SELENBP1 antibody (1:100) at room temperature for 1 h. Secondary antibody from the Envision HRP kit (Dako, CA) was allowed to incubate for 30 min. Staining development was performed with diaminobenzidine (DAB). Slides were then counterstained with hematoxylin, dehydrated with an ascending series of ethanol, cleared in xylene, and mounted. Staining intensity of each tissue core was quantified by using Quantity One 1-D analysis software (Bio-Rad, Hercules, CA, U.S.A.). The average intensity scores of 2 to 4 replicate cores from each case were obtained, and the sections was classified as negative (–), weak (+), moderate (++), and strong staining (+++) according to the scores.

### 2.8. Statistical Analysis

Statistical analysis was conducted with student’s t-test for independent groups (two-tailed) on all matched pairs of subjects. Chi-square tests were applied to study the relationship between *SELENBP1* expression and clinicopathological factors, such as tumor grade, viral status, and gender. The level of significance was taken as *p* < 0.05.

## 3. Results

### 3.1. SELENBP1 Was Downregulated in HBx-Expressing Cells

**Figure 1:**
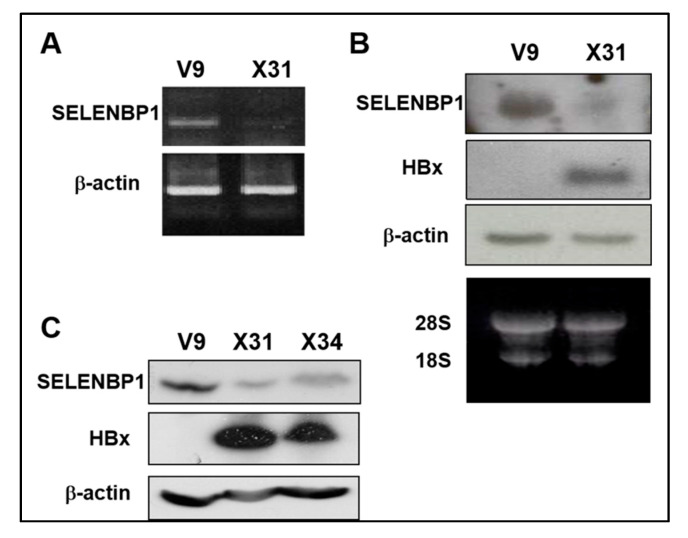
Reduced *SELENBP1* expression in *HBx*-expressing cells. (**A**) Expression of *SELENBP1* mRNA was analyzed by semi-quantitative RT-PCR analysis. RT-PCR was carried out on total RNA obtained from Chang V9 and Chang X31 cells using *SELENBP1* specific oligonucleotides. (**B**) The *SELENBP1* and *HBx* mRNA levels in total RNA were assayed by Northern blot analysis. (**C**) Whole cell lysates prepared from Chang V9, Chang X31, and Chang X34 cells were subjected to SDS-PAGE and immunoblotted using anti-SELENBP1 and anti-HA.

Chang V9 cells were used to study the effect of HBx on the *SELENBP1* promoter. Chang X31 and X34 cells [29] were used for positive controls constitutively expressing the HA-HBx protein. We investigated differential expression patterns of the cell lines by microarray comparative analysis and found that SELENBP1 is markedly decreased in HBx-expressing cells (Table S1). Based on the preliminary result, downregulation of *SELENBP1* mRNA expression by HBx is confirmed by semi-quantitative RT-PCR (Figure 1A) and Northern blot analysis (Figure 1B). Furthermore, Western blot analysis shows that SELENBP1 and HBx proteins are also in inverse proportion, consistent with cDNA microarray data (Figure 1C).

### 3.2. SELENBP1 Promoter Is Repressed by HBx in Dose-Dependent Manner

To examine the effect of HBx on the inhibition of *SELENBP1* gene expression, reporter plasmids in which the *Lck* coding sequence was driven by the *SELENBP1 promoter* were constructed. Chang V9 and HEK293 cells were co-transfected with the reporter plasmid (1584-Lck) and HBx expression plasmid (pRcCMV-HBx). Plasmid Myk-eGFP, expressing enhanced green fluorescence protein (eGFP) under murine cytomegalovirus (mCMV) immediate early gene promoter, was included in all transfection mixtures to normalize the transfection efficiency, and pcDNA3.1 was used as a negative control.

**Figure 2:**
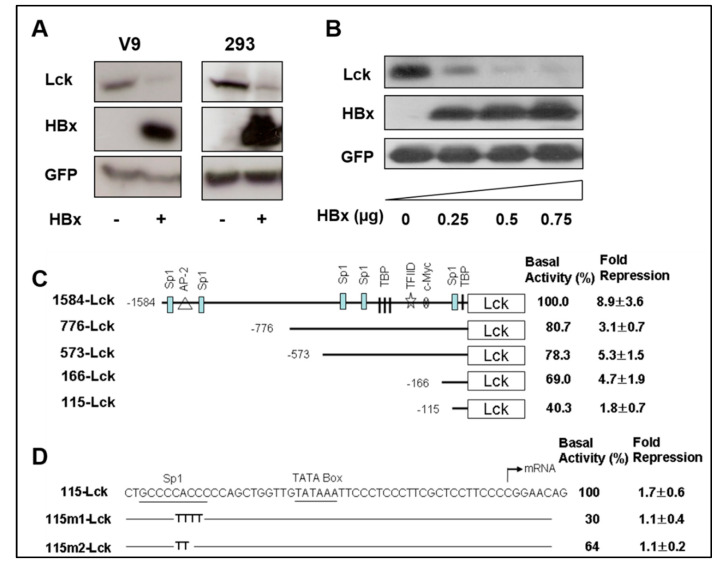
Repression of the *SELENBP1* promoter by HBx. (**A**) *HBx* expression plasmid or control plasmid was co-transfected into Chang V9 and HEK293 cells with *Lck* plasmid. Plasmid Myk-eGFP was used for normalizing the transfection efficiency, and pcDNA3.1 was used as a negative control. Two days after transfection, cell lysates were analyzed by Western blotting for investigation of the HBx-mediated down-regulation of *SELENBP1* promoter. (**B**) HEK293 cells were co-transfected with 0, 0.25, 0.5, and 0.75 μg of pRcCMV-HBx and *Lck* expression reporter plasmid. Two days after transfection, expression levels of Lck, HBx, and GFP in the transfected cells were determined by Western blot analyses. Representative bands are shown after 3 independent experiments. (**C**) The 1584-bp length *SELENBP1* promoter and its truncated constructs are schematically shown, and positions of putative transcriptional binding factor sites in the *SELENBP1* promoter are presented. Promoter activity was determined by measuring the level of Lck protein expression using NIH Image J software. The basal activity of 1584-Lck is designated to be 100%, and each promoter activity from truncated forms of the promoter, in the absence of HBx, is shown as a value relative to this basal activity. Fold repression was calculated by comparing the relative promoter activity of HBx-expressing cells with the basal activity of the control. Results as described fold repression are means ± SEM of at least three independent experiments. (**D**) Mutant constructs are identical to the wild-type 115-Lck sequence with the exception of the sequences shown in Sp1 site of each mutant construct. These constructs were cotransfected with an effector plasmid into 293 cells, and relative promoter activity and fold repression were determined.

To determine the promoter activity, expression levels of Lck and GFP protein were measured by Western blot analysis in the absence or presence of *HBx* expression. Remarkably, the mCMV promoter (*GFP* expression) do not show any decreased activity, but the *SELENBP1* promoter (*Lck* expression) was significantly inhibited by *HBx* expression in Chang V9 and 293 cells (Figure 2A). The *Lck* expression was decreased in HBx dose-dependent manner which means that the promoter activity of SELENBP1 is suppressed by HBx (Figure 2B). The suppression of the *SELENBP1* promoter by HBx is promoter-specific as it shows no effect on mCMV-directed transcription of the *GFP* gene in the same cells (Figure 2A). We also confirm the specificity of the HBx-mediated inhibitory effect on *SELENBP1 promoter* with a different set of Luciferase-reporter plasmids (Figure S1). These results indicate that the *SELENBP1* promoter is downregulated by HBx protein in a dose-dependent manner. Interestingly, mouse *Selenbp1* promoter as well as human *SELENBP1* promoter also show decreased expression strength by HBx. In a preliminary experiment performed with a HBx transgenic mouse model in our lab, we observed a decreased amount of SELENBP1 in the liver tissues that progressed to hyperplasia. Based on the observation, we examined whether HBx protein represses the expression of the mouse *Selenbp1* promoter. As expected, we confirm the down regulation of mouse *Selenbp1* promoter by HBx protein by luciferase reporter assay (Figure S1). In an empirical study investigating nucleotide sequence alignment using T-Coffee server (http://tcoffee.crg.cat), the sequences (−1~−160 nt) of the mouse *Selenbp1* promoter region show 75% similarity as compared with those of the human *SELENBP1* promoter region.

To identify the HBx-responsive region in the *SELENBP1* promoter, 5′ deletions of the promoter were constructed. Deletion constructs containing different lengths of the 5′-flanking sequences were co-transfected with either pcDNA3.1 or pRcCMV-HBx into 293 cells. Lck protein expression was measured by Western blot after transfection and promoter activity was determined by the level of Lck protein expression. There was a gradual decrease in promoter activity when upstream sequences were deleted (Figure 2C). Both basal promoter activity and fold suppression of each deletion construct were less than those of the longest promoter construct, 1584-Lck. 1584-Lck construct was suppressed approximately 9-fold by HBx, but 776-Lck suppressed only 3.1-fold. The basal promoter activity and fold suppression of 573-Lck (5.3 ± 1.5) and 166-Lck (4.7 ± 1.9) were similar to those of 776-Lck (8.9 ± 3.6). 115-Lck construct showed a 1.8-fold decrease. These results indicate that the *SELENBP1* promoter contains multiple regions responsible for suppression by HBx.

As 115-Lck, the smallest truncated promoter construct, was still HBx-responsive, we assumed that the Sp1 binding site at position −110 could be a candidate responsible for HBx-mediated suppression. In order to elucidate whether the Sp1 binding site in the *SELENBP1* promoter is necessary for HBx-mediated inhibition, Sp1 binding site mutants were generated using site-directed mutagenesis. When each mutant construct, containing two or four consecutive mutated bases, was co-transfected with HBx-expression vector, HBx-dependent suppression was maintained in 115-Lck construct, but not in plasmids 115m1-Lck and 115-m2-Lck (Figure 2D). Additionally, the sequence analysis (using a transcription factor binding site prediction tool, ConTra v3, http://bioit2.irc.ugent.be/contra/v3/#/step/1) of the mouse *Selenbp1* promoter region shows two Sp1 binding sites at −45 to −56 and −58 to −69 nt sites. These results suggest that Sp1 binding site at position −110 might be one of the HBx-responsive elements in the *SELENBP1* promoter. However, we cannot rule out the possibility that there are another HBx-responsive elements that mediate the HBx-dependent suppression of the *SELENBP1* promoter. In order to examine the responsiveness of SP1 binding site, in terms of the non-statistical significance of 115-Lck mutation analysis, it is necessary to additionally perform a promoter mutation assay using the 166-Lck plasmid.

### 3.3. SELENBP1 Protein Level Was Decreased in Hepatocellular Carcinoma

**Figure 3:**
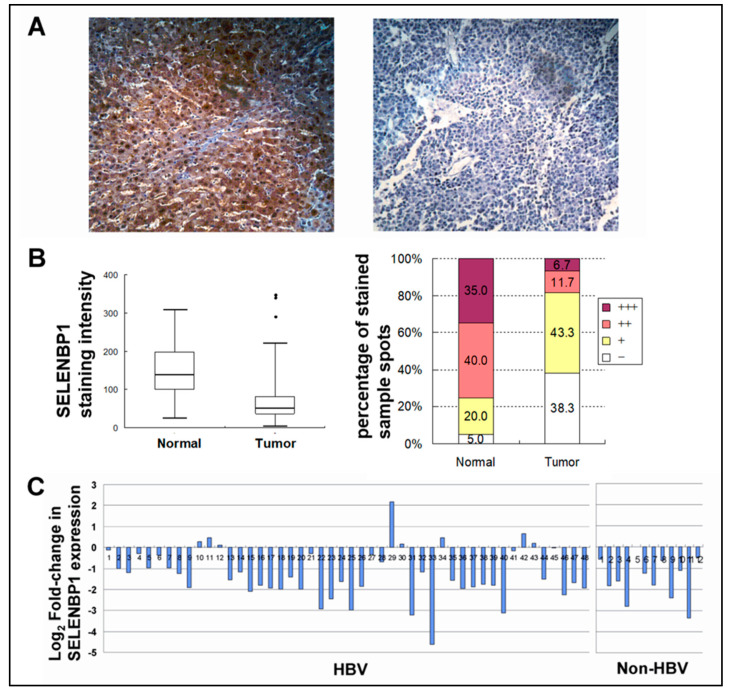
*SELENBP1* expression in normal and hepatocellular carcinoma (HCCs). (**A**) Representative images of SELENBP1 immunohistochemistry in non-tumor normal liver tissue (left panel) and matched liver tumor tissue (right panel) (Zeiss, x200). (**B**) Box plot showing the staining intensity of SELENBP1 in normal and tumor tissues (left panel). Percentages of strong (+++), moderate (++), weak (+), and negative (-) staining are depicted (right panel). Expression level of *SELENBP1* is significantly reduced in tumor tissues as compared with counterpart normal liver tissues. (**C**) The graph represents the levels of fold-change measured against matched non-tumor liver tissue. The level of expression is shown in log_2_–fold-changes. Each bar represents each individual patient tissue array of 60 pairs of HCC and their matched non-tumor liver tissues on microarray.

To evaluate the *SELENBP1* expression in hepatocellular carcinoma (HCC), 60 pairs of HCC and their matched non-tumor liver tissues on microarray were analyzed by immunohistochemistry. High *SELENBP1* expression was observed in normal liver tissues while the expression levels of *SELENBP1* were reduced in most tumor sections on the tissue microarray (Figure 3A). To determine the level of decrease of *SELENBP1* expression in HCC as compared with matched non-tumor (counterpart normal) liver tissues, the overall intensities of SELENBP1 staining were scored for quantification by using Quantity One 1-D analysis software (Bio-Rad, CA). In this experiment, the mean staining intensity scores in tumor tissues show a 2-fold decrease in scores as compared with matched non-tumor (counterpart normal) liver tissues (*p* < 0.0001) (Figure 3B). Seventy-five percent of normal liver tissues exhibit moderate (++) to strong (+++) expression of *SELENBP1*, whereas counterpart tumor tissues show moderate to strong at much lower rates (18.3%) (Figure 3B). The fold changes in *SELENBP1* expression in tumors against normal tissues of each individual samples are shown in Figure 3C. As compared with matched non-tumor (counterpart normal) liver tissues, levels of SELENBP1 were decreased in 43 HCC cases (71.7%) and were elevated in three cases (3.3%) more than 1.5-fold. In the other 15 (25%) couples of HCC and matched non-tumor (counterpart normal) liver tissues, no obvious difference of *SELENBP1* expression was detected. Other clinicopathological factors, such as information on the diagnosis of hepatitis B virus infection, gender, and tumor grades, had no relevance with *SELENBP1* expression (Table S2).

**Figure 4:**
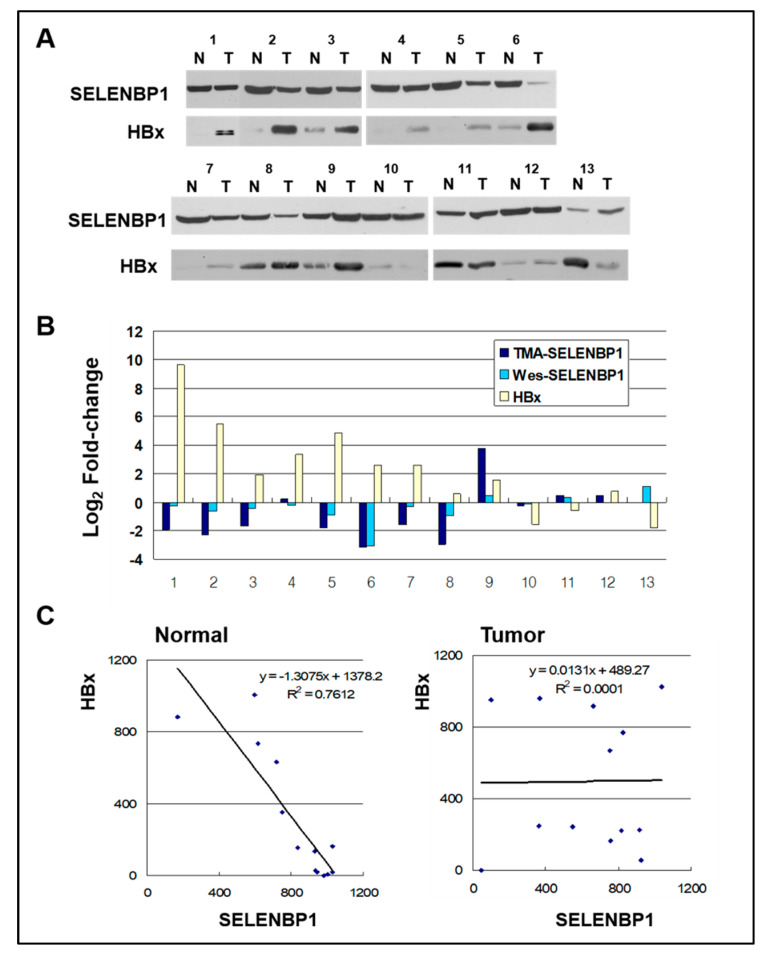
SELENBP1 and HBx in normal and HCCs. (**A**) Sixty micrograms of total protein from normal (N) and tumor (T) liver tissues of HCC patients with hepatitis B virus (HBV) were separated by SDS-PAGE and transferred to a membrane. The membranes were probed using anti-SELENBP1 and anti-HBx. (**B**) Comparison of changes in SELENBP1 protein expression level as measured by TMA and Western blot. Fold changes in HBx protein expression measured by Western blots are also shown. Values are expressed in fold change (Log_2_) compared to normal tissues. (**C**) Relationship between SELENBP1 and HBx protein levels as determined by linear regression analysis in normal and tumor tissues. A significant strong correlation was observed between SELENBP1 and HBx expression in normal tissues, and no correlation in tumor tissues. Regression equations and lines are shown in the graph.

To confirm the suppressed *SELENBP1* expression in HCC at protein level, 13 pairs of HCC and their matched non-tumor liver tissue lysates were analyzed by Western blotting (Figure 4A). The overall pattern of the Western blot is consistent with immunohistochemistry of the tumor tissue array in that SELENBP1 Western blot bands of tumor arrays tended to be more intensive than those of the matched non-tumor array (Figure 4B). In 10 (out of 13) cases with elevated HBx in tumors, 80% (8/10) show reduced SELENBP1 protein expression in Western blot, and 70% (7/10) show consistent results with immunohistochemistry of tumor tissue array. In the other three (out of 13) cases with reduced *HBx* expression, 66.6% (2/3) show elevated *SELENBP1* expression. Thus, there is an inverse relationship between SELENBP1 and HBx protein expression changes in about 69.2% (9/13) of the examined cases. Linear regression analysis was performed on non-tumor and matched tumor tissues to examine the causal relationship between SELENBP1 and HBx (Figure 4C). Regression analysis indicates a strong negative association between the amounts of SELENBP1 and HBx in matched non-tumor normal liver tissues (R^2^ = 0.7612), but no correlation in tumor tissues (R^2^ = 0.0001). These results suggest that HBx-mediated downregulation of *SELENBP1* expression might be involved in the early stages of HCC development process.

## 4. Discussion

Several studies demonstrated that HBx is capable of transcriptional suppression. HBx has been shown to downregulate the expression of p53 [38], XPB (p89, ERCC3) and XPD (p80, ERCC2) [51], PTEN [52], and p21 [53]. HBx also suppresses the expression of GST alpha, a detoxification enzyme, and selenoprotein P, known to be an antioxidant protein [54,55]. Changes in these factors may contribute to carcinogenic effects on liver cancers caused by the hepatitis B virus. We found that the mRNA level of SELENBP1 is reduced in HBx-expressing HeLa [Chang liver] cells using cDNA microarrays. In this study, we validated the evidence that HBx can downregulate the expression of *SELENBP1*. Reduced *SELENBP1* expression has been shown in multiple cancers. Being one of the most common cancers worldwide, the correlation of hepatocellular carcinoma (HCC) and SELENBP1 is under investigation. In our study, levels of SELENBP1 were significantly decreased in liver tumor tissues as compared with matched non-tumor (counterpart normal) liver tissues. Our finding, in terms of the decrease of SELENBP1 in tumor tissues, is consistent with the previous reports that described the suppression of *SELENBP1* in diverse types of epithelial cancers (details in introduction). However, there is little information on the molecular mechanisms inducing the downregulation of *SELENBP1* expression, except for epigenetic modification [7,19] and Nkx2-1-directed regulation [14].

As HBx is one of the causative agents of HCC, we examined the relationship between SELENBP1 and HBx expression in HBV-associated HCC. Western blot analysis shows that *SELENBP1* expression is negatively correlated with HBx in normal liver tissues of HBV-positive HCC patients (Figure 4A). This shows the in vivo relevance of *SELENBP1* downregulation by HBx. Interestingly, no correlations between SELENBP1 and HBx expression are found in tumor tissues (Figure 4C). Therefore, cellular processes resulting from the downregulation of *SELENBP1* expression in non-malignant cells may be involved in the early stage of HCC development and may be quite distinct from those in tumor cells. It could be speculated that in tumor cells a part of the regulatory process by HBx has likely been lost, or SELENBP1 might preferentially be regulated by other inhibitory mechanisms common in many cancers, such as epigenetic changes. The *SELENBP1* downregulation mechanism in tumor cells still remains to be identified. It is presumed that there are other mechanisms besides epigenetic changes suppressing the expression of *SELENBP1*, because previous reports showed that downregulation of *SELENBP1* is not related to promoter methylation or gene deletion in colon and lung carcinomas [7,19].

Interestingly, we observed that 3 cases out of 13 liver tumor/normal tissue pairs show decreased levels of HBx in tumor tissues as compared with matched non-tumor tissues (Figure 4). While in the remaining 10 cases, the expression level of *HBx* was increased in tumor tissues. The non-tumor tissue may also contain cells, residing in cirrhosis or chronic hepatitis lesions, that were already infected with HBV and are producing HBx protein. In addition, tumor tissue may cause a decrease in HBx expression due to mutations in the HBx gene integrated into the chromosome and the chromosome instability that are frequently observed in HCC [56]. This suggest that in some cases, HBx protein expression in non-tumor tissue may be higher than that in tumor tissue.

It is controversial that the *SELENBP1* expression pattern is not different between HBV and non-HBV (alcohol or hepatitis C virus or unknown) cases. However, the non-HBV sample number was small (12 cases) and it is possible that the unknown samples may express HBx, or HBx expression levels in some HBV-positive samples might be too low to affect *SELENBP1* expression. Further studies are needed to determine the cause of the decrease in *SELENBP1* expression in non-HBV liver cancer tissues.

The suppression of *SELENBP1* in many other cancers was suggested to be involved in tumorigenesis and poor prognosis. The fact that HBx can downregulate *SELENBP1* expression and that *SELENBP1* is suppressed in HCC suggests that the downregulation of *SELENBP1* by HBx may be a causative factor in the progression of HBx-associated HCC. There have been numerous reports describing a relationship between dietary selenium intake and risk of cancer [57,58]. Inverse correlation between plasma selenium levels and HCC was also demonstrated [59]. Dietary selenium supplementation showed a protective effect against HBV infection and HCC, indicating that selenium deficiency is associated with liver disease and HCC [60]. Despite evidence linking selenium or SELENBP1 deficiency to different disease conditions, the relationship between *SELENBP1* expression and selenium level is not yet known. It remains to be determined if *SELENBP1* downregulation by HBx can affect selenium level and make a patient with lower natural levels of selenium have a greater risk, due to the threshold being reached sooner. Future clinical trials could include changing dietary selenium levels and analyzing its effect on patients with early stages of HCC who have the different levels of SELENBP1.

Hepatitis B virus (HBV) infection accounts for at least 50% of cases of HCC worldwide. Indeed, HBV infection accounts for nearly 70% of hepatocellular carcinoma cases in Korea [61]. To the best of our knowledge, this is the first study that examined the correlation of a viral key protein and *SELENBP1* expression in HBV-associated hepatocellular carcinoma.

In conclusion, our study suggests that although the function of SELENBP1 is not known in the process of HCC development, *SELENBP1* downregulation by HBx protein may contribute to the carcinogenesis of HBV-associated HCC and be a potential event in HBV pathogenesis.

## Supplementary Materials

The following are available online at https://www.mdpi.com/1999-4915/12/5/565/s1, Figure S1: Luciferase reporter assay for inhibition of human and mouse *Selenbp1* promoter by HBx., Table S1: Genes downregulated in Chang X31 cells, cDNA microarray, Table S2: Relationship between *SELENBP1* expression and clinicopathological factors.
