# Supplementary material for: Hepatitis B Virus-X Downregulates Expression of Selenium Binding Protein 1
Source: Viruses. 2020 May 20;12(5):565. doi: 10.3390/v12050565 (PMC7291177; doi:10.3390/v12050565)
Supplement: Supplementary file 1 [file viruses-12-00565-s001.zip › Table S2.pdf]

Table S2. Relationship between SELENBP1 expression and clinicopathological factors

	Intensity of SELENBP1 staining				No. of cases	P-value
	-	+	++	+++		
**Normal**						
Virus						
HBV	2	9	19	18	48	0.811
Non-HBV	1	3	5	3	12	
Gender						
Male	1	11	21	16	49	0.086
Female	2	2	2	5	11	
Grade						
2	2	3	14	13	32	0.751
3	1	4	8	7	20	
**Tumor**						
Virus						
HBV	16	22	6	4	48	0.383
Non-HBV	7	4	1	0	12	
Gender						
Male	18	22	5	4	49	0.633
Female	5	4	2	0	11	
Grade						
2	13	12	4	3	32	0.888
3	7	9	3	1	20	

Normal and tumor samples from HCC patients were analyzed for SELENBP1 expression. Samples were categorized and analyzed according to clinicopathological factors, such as hepatitis virus, gender and tumor grades. Expression level of SELENBP1 had no correlation with clinocopathological factors of HCC. Chi-square tests were applied and the level of significance was taken as $p<0.05$.
