# Supplementary material for: Hepatitis B Virus-X Downregulates Expression of Selenium Binding Protein 1
Source: Viruses. 2020 May 20;12(5):565. doi: 10.3390/v12050565 (PMC7291177; doi:10.3390/v12050565)
Supplement: Supplementary file 1 [file viruses-12-00565-s001.zip › Fig. S1.docx]

**
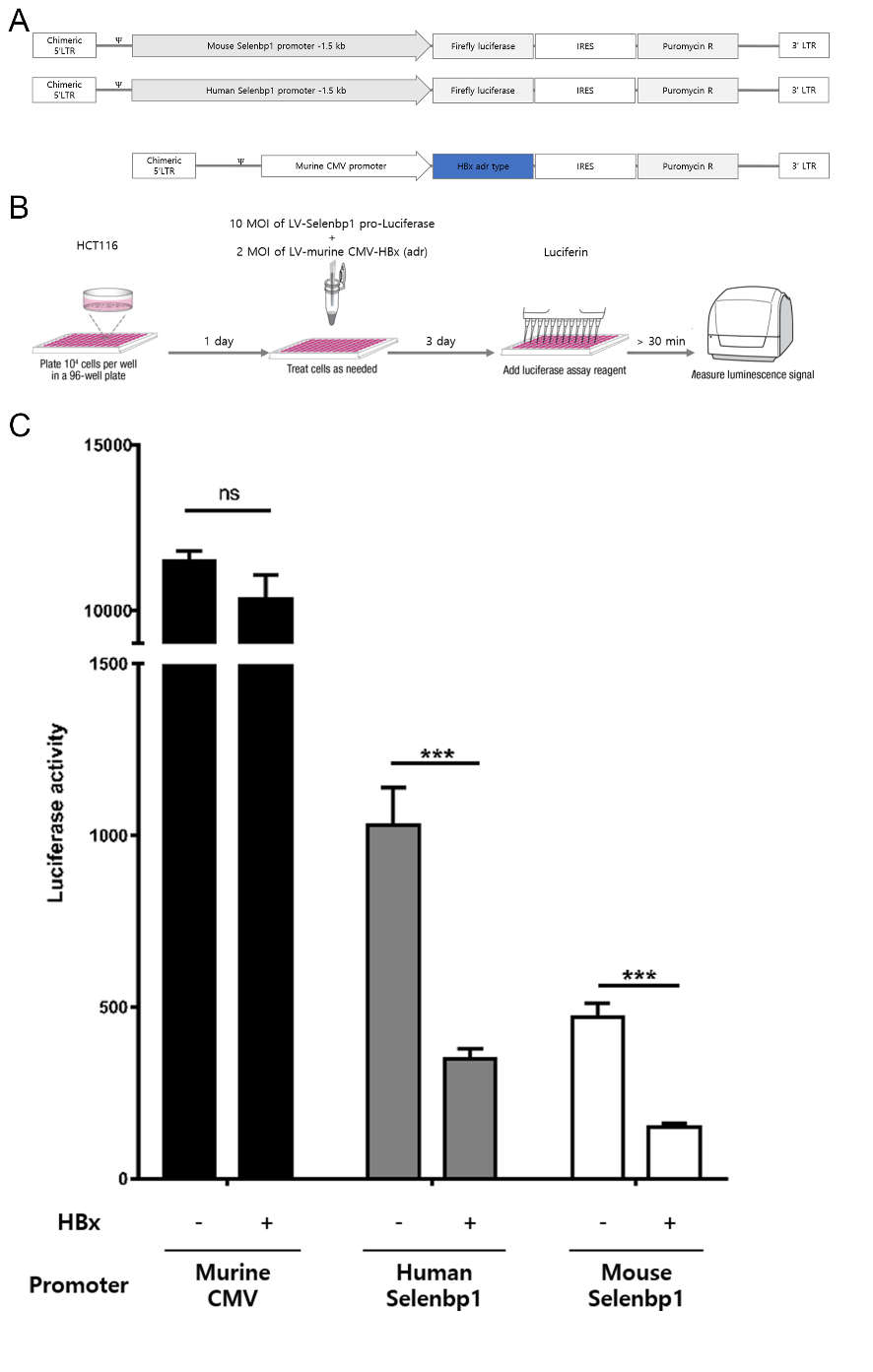
**

**Figure S1. Luciferase reporter assay for inhibition of human and mouse *Selenbp1* promoter by HBx.**

A. Constructs of lentiviral luciferase reporter system.

B. Illustration of the procedure for reporter assay

C. Transcriptional downregulation of *Selenbp1* promoters by HBx

Recombinant lentiviral vectors encoding firefly luciferase under mouse/human Selenbp1 promoter (Selenbp1 promoter: 1.5kb-Luc) and recombinant lentiviral vector encoding HBx (adr type) driven by murine CMV immediate-early promoter were produced and stored at -80℃. Human colorectal cancer cell line HCT116, a SELENBP1-negative cell line, was plated on 96-well plate 1 day before transduction. Ten MOI of lentiviral vectors encoding luciferase gene and 2 MOI of lentiviral vector encoding HBx were co-transduced to HCT116 cells with 8 μg/ml polybrene for 8 h. Three days after transduction, the transduced HCT116 cells were allowed to be equilibrated to room temperature for 15 minutes. Luciferin solution (100μl/well, Firefly Luc One-Step Glow Assay, 16196, Thermo, USA) was added, and the plate was incubated at room temperature for 4 minutes. The luminescence signal was detected by a luminometer (HTS Multi-Label Reader, Perkin Elmer, USA).

The lentiviral luciferase vector, LV-murine CMV-luciferase, was used as a control. The repression of transcription by HBx was promoter-specific because there was no inhibitory effect on murine CMV-directed transcription of the luciferase gene in the same cells. Three- fold decrease in luciferase activity of both human and mouse *Selenbp1* promoter was measured in HBx expressing HCT116 cells (Unpaired t test t=6.026, df=6, p=0.0009 in human SELENBP1 promoter, Unpaired t test t=7.542, df=6, p=0.0003 in mouse *Selenbp1* promoter. This result suggests that the HBx induces the transcriptional repression of the *Selenbp1* promoter.
