# Supplementary material for: Hepatitis B Virus-X Downregulates Expression of Selenium Binding Protein 1
Source: Viruses. 2020 May 20;12(5):565. doi: 10.3390/v12050565 (PMC7291177; doi:10.3390/v12050565)
Supplement: Supplementary file 1 [file viruses-12-00565-s001.zip › Table S1.pdf]

Table S1. Genes downregulated in Chang X31 cells, cDNA microarray

Gene	Description	NCBI #	1	2
POR	P450(cytochrome) oxidoreductase	Hs. 167246	-4.9	-4.0
PSME1	Proteasome activator subunit 1(PA28 alpha)	Hs. 75348	-3.2	-2.8
PGM1	Phophoglucomutase 1	Hs. 1869	-3.0	-3.0
ZNF76	Zinc finger protein 76	Hs. 29222	-3.0	-2.3
FBLN2	Fibulin 2	Hs. 198862	-4.6	-3.9
SELENBP1	Selenim binding protein 1	Hs. 334841	-4.6	-4.0
TUBA1	Tubulin, alpha 1(testis specific)	Hs. 75318	-2.4	-2.2
PGM1	Phosphoglucomutase 1	Hs. 1869	-3.7	-2.6
MGST3	Microsomal glutathione S-transferase 3	Hs. 111811	-3.1	-2.8
MOV10	Mov10, Moloney leukemia virus 10, homolog(mouse)	Hs. 20725	-3.2	-2.3
CKMT1	Creatine kinase, mitochondrial 1 (ubiquitous)	Hs. 153998	-2.2	-2.6
CHN2	Chimerin (chimerin) 2	Hs. 286055	-2.2	-2.4
P100	EBNA-2 co-activator (100kD)	Hs. 79093	-2.6	-5.2
RXRA	Retinoid X receptor, alpha	Hs. 20084	-2.0	-2.6
RPS10	Ribosomal protein S10	Hs. 76230	-2.6	-3.0

Chang (normal liver cell) and Chang X31 (Chang cells transfected with pRcCMB-HBx, stably expressing HBx) were compared. RNAs were prepared with RNeasy Midi kit (Qiagen, CA, USA) and cDNA array was performed. Experiments were repeated twice using different RNAs, harvested independently. Downregulated genes related to reactive oxygen species (ROS) are listed above.
